# Standardized Patient Practices: Initial Report on the Survey of US and Canadian Medical Schools

**DOI:** 10.3885/meo.2009.F0000208

**Published:** 2009-06-29

**Authors:** Lisa D. Howley, Gayle Gliva-McConvey, Judy Thornton

**Affiliations:** * Carolinas Medical Center, Charlotte, North Carolina; † Eastern Virginia School of Medicine, Virginia Beach, Virginia; ‡ University of Colorado, Denver

**Keywords:** Standardized patient, training program, educational activities, methodology, survey

## Abstract

**Background:**

There is currently a lack of information about the ways in which standardized patients (SPs) are used, how programs that facilitate their use are operated, the ways in which SP-based performance assessments are developed, and how assessment quality is assured. This survey research project was undertaken to describe the current practices of programs delivering SP-based instruction and/or assessment.

**Method:**

A structured interview of 61 individual SP programs affiliated with the Association of Standardized Patient Educators (ASPE) was conducted over a 7-month period. A web-based data entry system was used by the 11 trained interviewers.

**Results:**

The two most common reported uses of SPs were learner performance assessment (88% of respondents) and small-group instruction (84% of respondents). Fifty-four percent of programs hired 51–100 SPs annually and paid an average of $15 and $16 per hour for training time and portraying a case, respectively. The average reported number of permanent program employees, excluding SPs and temporary staff, was 4.8 (sd = 3.6). The most frequently reported salary range was $30,001–$45,000.

**Conclusion:**

We intend for these preliminary results to inform the medical education community about the functions of SPs and the structures of programs that implement these complex educational endeavors.

Since the development of the standardized patient (SP) in the early 1960s, the vast majority of medical schools in the United States and Canada have adopted this modality to instruct students, assess curricula, and/or certify skills.^1^ The practice has become so widespread that the National Board of Medical Examiners (NBME) recently added an SP-based performance examination to its required certification process for all US medical doctors: the United States Medical Licensing Exam (USMLE) Clinical Skills Examination.^2^ Although widespread, the use of SPs in the medical curricula seems to vary from informal and sporadic to formal and systematic. There is currently a lack of information regarding the ways in which SPs are used, how programs that facilitate their use are operated, how performance assessments are developed and how their quality is assured. Without such information, we are unable to investigate and establish best practices, develop standards for program operations, and continually improve our educational endeavors. In 2007, the Association of Standardized Patient Educators (ASPE) initiated a survey research project to fill this information gap for a better understanding of the uses of SPs in medical education. The goal of this survey research project was to describe the current practices of programs that deliver SP-based instruction and/or assessment. The primary research question was *“What are the functions of SPs and the structure of SP Programs in the US and Canada?”*

## Methods

### Interview Protocol

A structured interview protocol was developed by a small committee led by an educational researcher (LH) with expertise in survey design and administration. The structured interview method was chosen due to the nature of the questions and the complexity of the SP programs. A protocol was developed for conducting the structured interviews that included detailed instructions, an interview script, and an opportunity to provide notes regarding the process. The protocol was made available as a computer-assisted telephone interview. A web-based data entry system (WebSP™) was used to direct the interview and collect data. A total of 204 questions were included, divided into the 10 sections described in Table 1. The structured interview was developed to last up to 90 minutes. A separate 13-item interview protocol was developed for use if the institution did not have a formal SP Program. For purposes of this research, “standardized patient” was defined broadly as a layperson trained to instruct health care providers in communication and/or physical examination skills or a person who has been trained to portray a patient scenario for the instruction, assessment and/or practice of communication and/or examination skills of health care providers. A formal SP program was defined as an institution with one or more full time staff dedicated to the recruitment, training, and administration of SP-related activities.

**Table 1.** Interview Protocol Information

Category	Number of Items	Sample Content
Tracking	10	Dates and times for preliminary and primary calls
Preliminary information	2	Category of institution and geographic location
Participant Information	9	Details regarding interviewee including title, experience and education level, demographic information, job responsibilities
General Information	36	Details regarding SP Program (separate questions were asked to those without a formalized program) including year established, number of staff, website, nature of services provided, type of learners, etc.
General Program Operations	41	More specific details regarding program operations including number of SPs hired annually, contact hours, type of services offered and level of learner, terminology used, assessment details, case development process, use of data, methods of quality control and evaluation, policies and procedures, etc.
Space	13	Details regarding facilities for operations including whether space is dedicated, number and size of rooms, types of video and computer technologies, etc.
SP Recruitment & Training	49	Methods used to recruit and train SPs and raters, diversity of SP pool, hiring methods
Budget	11	Details regarding program finances including how much SPs are paid, whether funds are available for travel, research, etc.
Staff	30	Ten questions were asked of up to three separate staff members. These questions included status, title, education and background, job responsibilities, and annual salary. If the program has more than three staff members (in addition to the interviewee), the participant was to choose three diverse positions.
Final Questions	3	These questions pertained to outstanding and professional development needs of the program. Participants also had the opportunity to ask questions or make general comments.

### Sample and Sampling Frame

The sampling frame was the 2005 Association of Standardized Patient Educators (ASPE) membership database. The unit of analysis was the institution, and the interviewee was a single representative from that institution who could provide accurate and detailed information about the use of SPs. An attempt was made to reach every ASPE-affiliated institution. However, the decision to participate was completely voluntary, and we were able to sample 61 of 117 affiliated institutions (response rate = 52%). Reasons for non-participation included inability to identify appropriate institutional representatives, scheduling conflicts between the interviewer and participant, and the interview length. A list of participating institutions is provided in Table 2.

**Table 2.** Participating Institutions

A.T. Still University of Health Sciences, College of Osteopathic Medicine, AZ	University of British Columbia Faculty of Medicine, BC
Albany Medical College, NY	University of California San Diego School of Medicine, CA
Baylor College of Medicine, TX	University of Chicago Pritzker School of Medicine, IL
Des Moines University Osteopathic Medical Center, IA	University of Cincinnati College of Medicine, OH
Eastern Virginia Medical School, VA	University of Colorado School of Medicine, CO
Florida State University College of Medicine, FL	University of Connecticut School of Medicine, CT
George Washington University School of Medicine, DC	University of Illinois College of Medicine, IL
Johns Hopkins University, MD	University of Iowa Carver College of Medicine, IA
Keck School of Medicine of the University of Southern California, CA	University of Manitoba, Manitoba
Lake Erie College of Osteopathic Medicine-Bradenton, FL	University of Maryland, MD
Medical College of Georgia School of Medicine, GA	University of Massachusetts Medical School, MA
Medical College of Wisconsin, WI	University of Michigan Medical School, MI
Meharry Medical College, TN	University of Mississippi School of Medicine, MS
Mercer University School of Medicine, GA	University of Nebraska College of Medicine, NE
Midwestern University AZ College of Osteopathic Medicine, AZ	University of New Mexico, NM
Northeastern Ohio Universities College of Medicine, OH	University of North Dakota, ND
Northwestern University Feinberg School of Medicine, IL	University of Oklahoma College of Medicine, OK
Nova Southeastern University College of Osteopathic Medicine, FL	University of Ottawa, ON
Ohio State University, OH	University of Pennsylvania School of Medicine, PA
Penn State University College of Medicine, PA	University of Texas Health Science Center at San Antonio, TX
Philadelphia College of Osteopathic Medicine, PA	University of Texas Medical Branch at Galveston, TX
Queen's University, ON	University of Texas Medical School at Houston, TX
Southern Illinois University School of Medicine, IL	University of Utah School of Medicine, UT
St. Louis University School of Medicine, MO	University of Vermont College of Medicine, VT
Stanford University School of Medicine, CA	University of Virginia School of Medicine, VA
Stony Brook University School of Medicine, NY	University of Washington School of Medicine, WA
Temple University School of Medicine, PA	Vanderbilt University, TN
Tulane University School of Medicine, LA	Wayne State University School of Medicine, MI
UNC Chapel Hill School of Medicine, NC	Western University College of Osteopathic Medicine of the Pacific, CA
Uniformed Services University, MD	Yale University School of Medicine, CT
University of Arizona College of Medicine, AZ	

### Data Collection

Eleven volunteer members of ASPE's Standards of Practice Committee conducted structured interviews of representatives from osteopathic and allopathic medical schools in the United States and Canada. The volunteer intervewers were all experienced SP educators who trained to conduct the structured telephone interviews. The training was led by the primary author (LH) and one of her doctoral students in educational research. Topics included telephone interviewing do's and don't's as well as a detailed review of the interview protocol. A pilot test was conducted to evaluate the protocol and, if needed, further prepare the interviewers. Specifically, members of the ASPE Board of Directors were interviewed and provided feedback on the interviewer's style, content of the protocol, and overall satisfaction with the process. This information was used to further refine the protocol. Several items were revised and others were deleted to reduce the length of the interview.

Institutions were randomly assigned to interviewers. The representative (or interviewee) was also sent a list of questions in order to prepare for the interview. The interviewer then arranged a convenient time for the survey during a preliminary call. Procedures were in place to identify an alternate representative when the initial person was not able or not willing to participate. Verbal agreement to continue with the primary telephone interview constituted consent. Participants were told in writing and verbally that their participation was voluntary, that the information they provided would be kept confidential and that when reporting results from the survey only group data would be shared. When quotes would be used, any identifying information would be masked. Data were entered by the interviewer during the call using the electronic database WebSP™. Permission was granted and renewed for this study by the Institutional Review Board of the University of North Carolina at Charlotte.

## Results

**Figure 1.**
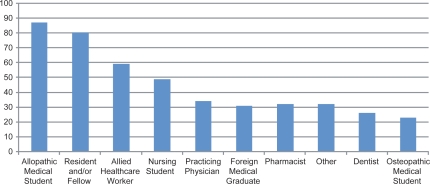
Percent of Programs Providing Services for Healthcare Practitioners (n = 55).

Sixty-one institutions participated in the survey. Due to technical difficulties, two interviews had to be repeated and four others were excluded from the analysis due to incomplete results. All but three institutions were located in the United States. See Table 3 for additional geographic information. All participants reported having a formal SP program defined as one or more full time staff persons dedicated to the recruitment, hiring, and/or training of SPs. The average age of SP programs was 13 years (SD = 6.5), with a range of 1 to 32 years. Seventy-four percent (n = 42) reported providing services for more than 1 institution or educational program. Figure 1 reports the types of learners these SP Programs reported serving.

**Table 3.** Percent of Participating Institutions by Geographic Region

Region	%age (n)
US – Northeastern	23% (14)
US – Southern	28% (17)
US – Central	25% (15)
US – Western	18% (11)
Canada	6% (4)

### Program Operations

**Figure 2.**
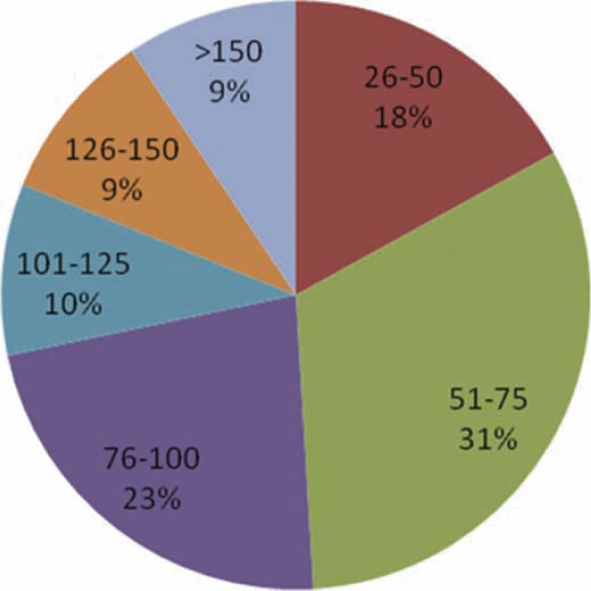
Number of SPs Hired Annually by Program (n = 53).

The largest percentage of programs reported hiring between 51–75 SPs annually (see Figure 2). SPs were used by the vast majority (n = 50, 88%) of programs for assessing learner performance and small group instruction (n = 48, 84%). A smaller majority also reported using SPs for class or lecture demonstration (n = 36, 63%) and individualized instruction (n = 32, 56%). Sixty-three percent (n = 36) of participants reported providing an SP-based assessment intended to prepare students to take a national board or certification examination (i.e., USMLE Clinical Skills Exam).

The majority of participants (n = 50, 88%) reported having developed SP cases internally for use within the program, while 72% (n = 68) reported having used one or more cases previously developed by an external institution. When asked about policies and procedures for the SP program, a majority of participants (n = 35, 66%) reported having no formal manual for program operations. 56% (n = 32) reported having no policy restricting the number of hours and/or sessions the SPs could work. Most participants (n = 48, 84%) reported that the performance of SPs (i.e., ability to portray a case, evaluate performance) within the program was evaluated for quality control purposes.

### Space

When asked about locations for SP activities, 86% (n = 49) reported having designated space available for program activities. 56% (n = 32) reported that this space was shared for purposes other than SP-related activities. Eighty-two percent (n = 47) of participants reported having a room or area from which encounters were directly monitored. About half of the interviewees (n = 22, 42%) reported that the average size of an examination room for SP encounters was 8 feet by 10 feet. Eighty-nine percent (n = 51) reported video recording SP encounters and of these, 88% (n = 45) reported having cameras mounted within the room/s. Twenty-nine percent (n = 14) recorded with VHS, 47% (n = 24) recorded digitally, and 24% (n = 12) used both technologies.

### SP Recruitment/Training

When asked about recruitment and training of SPs, the majority reported that physical examination (n = 42, 74%), references (n = 32, 60%), and background checks (n = 29, 55%) were not conducted prior to hiring applicants. Seventy-two percent (n = 41) of participants reported requiring SPs to sign legal waivers, agreements, or related documents before working (i.e., consent forms, work agreements). The average number of reported hours of training required before a new SP performed a role was 5.5 (SD = 5). Sixty-five percent (n= 37) reported that the average number of training hours was variable based on SP experience and type of encounter or activity, with more training required for less experienced SPs and encounters with higher stakes. The average hourly rate paid to SPs for various roles ranged from US$15 to US$48 (see Table 4).

**Table 4.** Average Hourly Wages Paid to SPs for Select Activities

SP Activity	Mean Hourly Rate (SD)	Median	Min-Max
Training (n = 42)	$15 (4.8)	15	$8–$25
Portraying a case with or w/o physical exam and evaluating performance (n = 50)	$16 (3.2)	15	$10–$25
Portraying a case with or w/o physical exam w/o evaluating performance (n = 48)	$16 (3.8)	15	$10–$30
Being examined and teaching non-invasive physical exam or communication skills (n = 48)	$17 (4.5)	17	$10–$35
Being examined and teaching invasive physical examination skills (n = 32)	$48 (28.2)	40	$16–$145

### Program Staff

The average reported number of permanent employees, excluding SPs and temporary staff, working within a program was 4.5 (SD = 3.8) (see Table 5). Participants were asked a series of standard questions about as many as 3 program staff members (permanent paid employees, including faculty and non-faculty, full and part time, but excluding SPs and/or temporary workers). When all full time staff members were combined (n = 123), the most frequently reported salary range was $30,001–$45,000. The minimum reported range was US$15,001–$30,000 and the maximum was >US$105,001. When differentiating salaries by geographic region, the most frequently reported salary range for the Western United States was $US45,001–$60,000. All other regions (including Canada) reported US$30,000–$45,000 as the most common salary range. Table 6 displays reported salaries for staff by education levels.

**Table 5.** Number of Permanent Staff* (n = 56)

Position	Mean	SD	Median	Min to Max Range
Full Time	2.7	2.0	2.0	0–12
Part Time	2.1	3.3	1.0	0–20
*Total*	*4.8*	*3.6*	*4.0*	*1–22*

*Permanent paid employees, including faculty and non-faculty, full and part time, and excluding SPs and/or temporary workers

**Table 6.** Staff Salary Range by Education Level (n = 112)

Education Level	Mode	Min to Max Range
Some College or AA (n = 17)	$30,001–$45,000	$15,001–$30,00 to $45,001–$60,000
Bachelors Degree or 4 years of College (n = 49)	$30,001–$45,000	$15,001–$30,00 to $60,001–$75,000
Masters Degree (n = 27)	$45,001–$60,000	$15,001–$30,00 to $75,001–$90,000
PhD or EdD (n = 11)	$60,001–$75,000	$60,001–$75,000 to >$105,001
MD or DO (n = 8)	>$105,001	$45,001–$60,000 to >$105,001

## Conclusions

These preliminary findings provide insight into current SP practices within 61 ASPE-affiliated institutions. Limitations of this survey research project include a weak response rate and underrepresentation of Canadian and osteopathic schools. Although this sample does represent 42% of all institutional members of the Association of American Medical Colleges (AAMC), these weaknesses limit our ability to generalize beyond the current sample of institutions. A related weakness includes our inability to provide data regarding reasons for non-participation.

According to our findings, these standardized patient programs were busy educational enterprises. The most common reported uses of SPs were the assessment of learner performance (88%) and small group instruction (84%). These programs provided opportunities for internal students and residents, as well as for external clients in the broader field of healthcare education. SP programs flourished in the 1990s, when 50% were first established. The majority of programs hired between 51–75 SPs annually and paid an average of $15 and $16 per hour for time spent training and portraying a case, respectively. This rate was significantly higher for encounters requiring the teaching and/or evaluation of invasive physical exam skills. The number of hours required prior to case portrayal varied greatly between 2 and 20 hours (M = 5.5, SD = 5). Policies and procedures with regards to SP employment and safety issues also varied between programs. Approximately 1 in 4 programs did not require SPs to sign consent forms or work agreements in relation to their employment within the program. Our data reflected that SP educators comprised a diverse group of professionals serving a variety of educational roles, including Directors, Coordinators, Trainers, Technicians, and Administrators. The most common degree among all SP educators was a bachelors degree (or 4 years of college) followed by a masters degree. As expected, salary ranges reflected this diversity and varied according to role and educational background.

The extent to which these varied practices impacts the quality of educational programming is to be determined. Further research regarding the use of SPs will be needed to better understand issues of training, case portrayal, and quality assurance. Additionally, questions regarding best practices for case development and administration remain unanswered. Some sample questions to be addressed include: (a) Are health screenings necessary for SPs? (b) What policies and procedures are necessary when using SPs to teach and evaluate learners? (c) How is SP quality assured? (d) What is the effect of time of portrayal on SPs (fatigue, accuracy rate, etc.)? (e) What is the impact of training time on SPs performance? (f) What training methods are optimal for SPs learning to portray a role/evaluate performance/provide feedback/etc.? (g) Does diversity in an SP pool matter? (h) What is the impact of case content on SP performance? Is there an optimal format for case materials? (i) What are the optimal qualities of an SP educator/trainer?

Although additional results from this project are forthcoming, we intend for these preliminary results to inform and guide the medical education community about the functions of SPs and the structures of programs which implement these complex educational endeavors. To date no such survey has been conducted which investigates the inner operations of SP programs. The current information affords us the opportunity to better understand and acknowledge the various SP practices in place. Our hope is that this information will stimulate further research and establish a foundation for setting program standards. As the use of SPs continues to expand and grow, additional research along with more transparent details regarding their function and structure will allow us to continually monitor, justify, and improve our educational endeavors.

## Notes

Contributing author (LH) currently serves as an Associate Editor to Medical Education Online. At the time this research was conducted, LH was a faculty member at the University of North Carolina at Charlotte and JT was a staff member at University of Texas Medical Branch in Galveston Texas.
